# Mitral valve repair and redo repair for mitral regurgitation in a heart transplant recipient

**DOI:** 10.1186/1749-8090-7-100

**Published:** 2012-09-29

**Authors:** Wobbe Bouma, Johan Brügemann, Inez J Wijdh-den Hamer, Theo J Klinkenberg, Bart M Koene, Michiel Kuijpers, Michiel E Erasmus, Iwan CC van der Horst, Massimo A Mariani

**Affiliations:** 1 Department of Cardiothoracic Surgery, University Medical Center Groningen, Groningen, the Netherlands; 2 Department of Cardiology, University Medical Center Groningen, Groningen, the Netherlands

**Keywords:** Heart transplantation, Mitral regurgitation, Mitral valve repair, Reoperation

## Abstract

A 37-year-old man with end-stage idiopathic dilated cardiomyopathy underwent an orthotopic heart transplant followed by a reoperation with mitral annuloplasty for severe mitral regurgitation. Shortly thereafter, he developed severe tricuspid regurgitation and severe recurrent mitral regurgitation due to annuloplasty ring dehiscence. The dehisced annuloplasty ring was refixated, followed by tricuspid annuloplasty through a right anterolateral thoracotomy. After four years of follow-up, there are no signs of recurrent mitral or tricupid regurgitation and the patient remains in NYHA class II. Pushing the envelope on conventional surgical procedures in marginal donor hearts (both before and after transplantation) may not only improve the patient’s functional status and reduce the need for retransplantation, but it may ultimately alleviate the chronic shortage of donor hearts.

## Background

Although cardiac transplantation has become a relatively common procedure, its major limitation is still the chronic shortage of donor hearts. Potentially, the donor pool could be expanded by extending donor selection criteria and by performing conventional surgical procedures on the donor heart, such as coronary artery bypass grafting or valve repair [1-4]. The limits of these procedures are being pushed to improve the patient’s functional status and to reduce the need for re-transplantation.

This report shows the feasibility of mitral valve repair and re-repair in a transplanted heart. To our knowledge, this is the first report of mitral valve re-repair in a transplanted heart.

## Case presentation

A 37-year-old man (length 1.98 m, weight 120 kg, body surface area 2.57 m^2^) with end-stage idiopathic dilated cardiomyopathy underwent an orthotopic heart transplant in June 2006. The donor was a 53-year-old man with no history of heart disease who had suffered intracerebral hemorrhage. A predonation echocardiogram revealed grade 1+ mitral regurgitation (MR), no other valvular lesions, and a moderate left ventricular (LV) function (Table 1). Coronary angiography was unavailable. Palpation did not reveal any coronary artery disease.

**Table 1:** Overview of events, treatment, NYHA class, and echocardiographic follow-up

**Event**	**Month & year**	**Treatment**	**Recipient NYHA class**	**TTE/TEE**	**Echocardiographic findings**
Heart transplant	June 2006	−	4	Pre-op donor TTE	MR 1+, TR 0, moderate LVF
Discharge	June 2006	−	3	Pre-discharge TTE	MR 2+, TR1+, moderate LVF (inferoposterior hypokinesia)
NSTEMI (inferoposterior)	June 2007	PCI RCA with 2 PRO-kinetic stents	3	−	−
Follow-up	January 2008	−	4	TTE and TEE	MR 4+ (Figure 1A), TR 2+, dilatation of the LV and deterioration of LVF
					MR severity: jet surface area (18.5 cm^2^)/ LA surface area (36.5 cm^2^) = 51%; vena contracta = 65 mm
					Mitral geometry: annular diameter = 39 mm (TEE, LAX), 40 mm (TTE, PLAX), 43 mm (TTE, AP4CH); intercommissural width = 36 mm (TEE, basal SAX); interpapillary muscle distance = 12 mm (TTE, PSAX); tenting height = 5 mm (TTE, AP4CH), 6 mm (TTE, PLAX); tenting area = 1.0 cm^2^ (TEE, LAX), 1.1 cm^2^ (TTE, AP4CH); posterior tethering angle = 20° (TTE, AP4CH); anterior tethering angle = 16° (TTE, PLAX)
NSTEMI (inferior)	May 2008	Mitral valve repair (CE classic 32 mm ring); hybrid PCI of the Cx	2	Post-op TTE	MR 1+, TR 1+, moderate LVF
Total AV block	May 2008	DDD-pacemaker	2	−	−
Follow-up	June 2008	−	3	TTE and TEE	MR 4+ (ring dehiscence) (Figure 1B), TR 4+ (Figure 1C), moderate LVF
	July 2008	Redo mitral repair (ring refixation) and tricuspid repair (CE classic 36 mm ring)	2	Post-op TTE	MR 1+, TR 1+, moderate LVF
	August 2008	−	2	TTE	Moderate LVF, intraventricular dyssynchronia
	September 2008	Upgrade to CRT-D	2	−	−
	July 2010	−	2	TEE	MR 1+ (Figure 1D), TR 1+ (Figure 1D), moderate LVF
	February 2012	−	2	TTE	MR 1+, TR 1+, moderate LVF

AP4CH, apical four-chamber view; AV, atrioventricular; CE, Carpentier-Edwards; CRT-D, cardiac resynchronization therapy-defibrillator; Cx, circumflex coronary artery; DDD, dual chamber/dual demand; LA, left atrial; LV(F), left ventricular (function); MR, mitral regurgitation; NSTEMI, non-ST-segment elevation myocardial infarction; NYHA, New-York Heart Association; PCI, percutaneous coronary intervention; (P)LAX, (parasternal) long-axis view; (P)SAX, (parasternal) short-axis view; RCA, right coronary artery; TEE, transesophageal echocardiography; TR, tricuspid regurgitation; TTE, transthoracic echocardiography.

The donor heart was preserved with St. Thomas’ solution (flush-perfusion) and implanted using the biatrial anastomotic technique. The total ischaemic time of the donor heart was 226 minutes. Intraoperative transesophageal echocardiography (TEE) (during dopamine and noradrenaline support after weaning from cardiopulmonary bypass) revealed grade 1+ MR. The postoperative course was complicated by prolonged inotropic support (3 days) and atrial fibrillation. The donor heart experienced substantial ischemic damage as shown by high levels of postoperative serum creatine kinase (maximum level 2500 U/L) and creatine kinase-MB (maximum level 246 U/L). A predischarge transthoracic echocardiogram (TTE) revealed grade 2+ MR, grade 1+ tricuspid regurgitation (TR), inferoposterior hypokinesia, and a moderate LV function. Sixteen routine endomyocardial biopsies in the first year after transplantation showed no significant rejection episodes (International Society of Heart and Lung Transplantation (ISHLT) grade ≤ IA).

**Figure 1:**
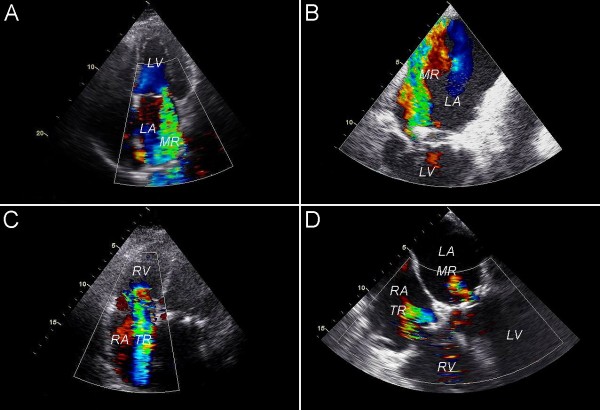
**Doppler echocardiographic imaging.A**. Preoperative TTE; grade 4+ MR. **B**. Postoperative TEE; grade 4+ recurrent MR along the posterior annulus. **C**. Postoperative TTE; grade 4+ TR. **D**. Two-year follow-up TEE; grade 1+ residual TR and MR. LA, left atrium; LV, left ventricle; MR, mitral regurgitation; RA, right atrium; RV, right ventricle; TEE, transesophageal echocardiography; TR, tricuspid regurgitation; TTE, transthoracic echocardiography.

In June 2007, the patient suffered an inferoposterior non-ST-segment elevation myocardial infarction (NSTEMI). A percutaneous coronary intervention (PCI) of the right coronary artery was performed with implantation of two Biotronik PRO-kinetic stents (Biotronik, Berlin, Germany). The circumflex coronary artery did not show any abnormalities. In January 2008, TTE and TEE revealed grade 4+ central MR (Figure 1A), grade 2+ TR, and a dilated LV with deterioration of the moderate LV function. Additional analysis of MR severity and mitral valve geometry is shown in Table 1. Mitral valve surgery was initially postponed, but in the following months complaints of dyspnea on exertion worsened and in May 2008 the patient suffered an inferior NSTEMI (this time based on a stenosis in the circumflex coronary artery). A hybrid treatment strategy was chosen with mitral valve surgery followed by PCI of the circumflex coronary artery.

In May 2008, mitral valve repair was performed through a redo median sternotomy. The mitral valve was exposed with a right atrial, trans-septal approach. No structural mitral valve abnormalities were found and saline testing revealed central regurgitation. MR was presumed to be ischaemic in etiology. After careful sizing, annular dilatation was corrected by implantation of an undersized 32 mm Carpentier-Edwards classic mitral valve annuloplasty ring with interrupted 2-0 Ticron sutures (i.e., a downsizing of two ring sizes). Postoperative recovery was complicated by a total atrioventricular (AV) block, which eventually required implantation of a DDD (dual chamber/dual demand)-pacemaker. On the seventh day after surgery, TTE showed grade 1+ MR and TR and a moderate LV function.

Follow-up TTE and TEE after six weeks showed severe recurrent MR (grade 4+) along the posterior annulus (Figure 1B) and severe TR (grade 4+) (Figure 1C). In July 2008, a right anterolateral thoracotomy was performed. The mitral valve was again exposed with a right atrial, trans-septal approach. Inspection revealed mitral annuloplasty ring dehiscence along the posterior annulus. The annuloplasty ring was refixated with interrupted 2-0 Ticron pledgeted sutures. Inspection of the tricuspid valve revealed no structural abnormalities. A 36 mm Carpentier-Edwards classic tricuspid annuloplasty ring was implanted. On the fifth day after surgery, TTE showed grade 1+ MR and TR and a moderate LV function.

In August 2008, TTE still showed a moderate LV function with evidence of intraventricular dyssynchronia. In September 2008, the DDD-pacemaker was upgraded to CRT-D (cardiac resynchronization therapy-defibrillator). In July 2010 (after a follow-up of two years) (Figure 1D) and in February 2012 (after a follow-up of nearly four years), MR and TR remained grade 1+, LV function remained moderate, and the patient remained in NYHA functional class II.

## Discussion

The reported experience with repair of atrioventricular valves after cardiac transplantation is still limited. Publications include different reports of single or double atrioventricular valve repair, either before (ex vivo bench surgery) or after transplantation [1-4]. However, this case is the first report of mitral valve re-repair in a transplanted heart.

Atrioventricular valve regurgitation after heart transplantation is common [1]. Early after transplantation, regurgitation is attributable to edema and poor lymphatic drainage, which normally subsides after about three months [1,4]. Some authors assume that the biatrial anastomosis results in atrioventricular size mismatch or malalignment, which lies at the basis of annular dilatation and valve dysfunction [1]. The bicaval anastomosis has been shown to preserve atrial geometry and size, and may be associated with reduced atrioventricular valve dysfunction [5]. Progression of natural valve disease in the donor heart at the time of transplantation may also cause atrioventricular valve regurgitation [1]. Endomyocardial biopsy-induced chordal damage is a known cause of TR [1] and accelerated graft atherosclerosis may lead to ischemic valve disease [1,3].

The etiology in this case is most likely chronic ischaemic MR (CIMR); the donor heart was subjected to a relatively long ischemic time, there was a documented inferoposterior myocardial infarction, inferoposterior hypokinesia, LV dilatation, and no structural mitral valve abnormality [6]. The posterior part of the mitral valve annulus has been shown to experience the largest amounts of mechanical strain during the cardiac cycle [7]. In addition, the biatrial method may have contributed to distortion of the atrial geometry with increased strain levels on the posterior annulus. Large amounts of strain on the sutures of the posterior part of the annuloplasty ring may eventually have caused dehiscence. Careful analysis of preoperative tenting parameters (Table 1) showed values well below the cutoff values of independent predictors of annuloplasty failure [6]. Since initial mitral annuloplasty was justified based on these parameters, we chose to perform mitral valve re-repair. Initial mitral valve annuloplasty with a more flexible ring (e.g., the Carpentier-Edwards Physio II ring) or a ring specifically tailored for CIMR (e.g., the Edwards GeoForm ring) might have reduced the chance of annuloplasty dehiscence. At the time, however, these rings were not yet in use or available on the market. The etiology of TR in this case remains uncertain. Since no structural abnormalities were found, it may be related to atrioventricular size mismatch, progression of natural valve disease, or a combination of both.

## Conclusions

In conclusion, double atrioventricular valve repair (including mitral valve re-repair) can be performed with good mid-term results after orthotopic heart transplantation. Acceptance of marginal donor hearts may lead to a complicated and demanding postoperative course, as shown in this case. However, pushing the envelope on convential surgical procedures in the transplanted heart may not only improve the patient’s functional status and reduce the need for retransplantation, but it may ultimately alleviate the chronic shortage of donor hearts.

## Consent

Written informed consent was obtained from the patient for publication of this case report and any accompanying images. A copy of the written consent is available for review by the Editor-in-Chief of this journal.

## Competing interests

The authors declare that they have no competing interests.

## Authors’ contributions

WB and JB collected the data and wrote the manuscript. IW, TK, BK, MK, ME, IH, and MM participated in the design of the manuscript and they revised and critically reviewed the manuscript. All authors read and approved the final manuscript.
